# Seroprevalence of *Neospora caninum* infection and associated risk factors in cattle in Shanxi Province, north China

**DOI:** 10.3389/fvets.2022.1053270

**Published:** 2022-11-29

**Authors:** Hui Cao, Wen-Bin Zheng, Yu Wang, Wen-Wei Gao, Qing Liu, Xing-Quan Zhu, Yu-Ping Lei, Bayaer Tumen, Hong-Yu Song

**Affiliations:** ^1^College of Veterinary Medicine, Shanxi Agricultural University, Jinzhong, China; ^2^Key Laboratory of Veterinary Public Health of Higher Education of Yunnan Province, College of Veterinary Medicine, Yunnan Agricultural University, Kunming, China; ^3^Veterinary Laboratory, Shanxi Provincial Animal Disease Prevention and Control Center, Taiyuan, China

**Keywords:** *Neospora caninum*, seroprevalence, cattle, ELISA, Shanxi Province, China

## Abstract

*Neospora caninum* is an obligate intracellular parasitic protozoan that can cause abortions in cattle and pose considerable economic losses to the cattle industry. As a major livestock province, little is known of *N. caninum* infection in cattle in Shanxi Province, north China. In order to investigate the seroprevalence of *N. caninum* in cattle in Shanxi Province, 978 cattle serum samples were collected from 11 cities in three representative geographical locations in Shanxi Province, and the *N. caninum*-specific IgG antibodies were examined using an indirect enzyme linked immunosorbent assay (ELISA) kit commercially available. The results showed that 133 of the 978 examined cattle serum samples (13.60%, 95% CI = 11.45–15.75) were positive for *N. caninum* antibodies, and the seroprevalence in different cities ranged from 0 to 78.89%. The geographical location and management mode were the risk factors associated with *N. caninum* infection in cattle herds in Shanxi Province. Cattle in Northern and Central Shanxi Province as well as cattle whose management mode is that of large-scale cattle farming companies are more susceptible to *N. caninum* infection. This was the first large-scale survey of *N. caninum* seroprevalence and assessment of associated risk factors in cattle in Shanxi Province, which provided baseline information for the prevention and control of *N. caninum* infection in cattle in Shanxi Province, north China.

## Introduction

*Neospora caninum* is an obligate intracellular parasitic protozoan that has a complex life cycle and infects a number of warm-blooded animals (1, 2). *N. caninum* can complete sexual reproduction in canids which is the definitive hosts of this parasite; while many important domestic and wild animals, such as cattle, sheep and camels, can serve as intermediate hosts (3–5). Because dogs often can move freely in the cattle herds, the risk of cattle infection with *N. caninum* is high, and the seroprevalence of *N. caninum* can be as high as 90% in some cattle herds (6, 7). *N. caninum* infection can cause abortion, stillbirths in cattle or neurological disorders in newborn calves (8, 9). The infection has considerable negative economic impacts on the cattle industry, due to abortions, premature cow culling and reduced milk production (10, 11).

Neosporosis is distributed worldwide, which has attracted the attention by the global livestock industry (12, 13). According to reports, the seroprevalence of *N. caninum* was 21.03% in dairy cows in Northern Greece, and 26.20% in cattle in Mongolia (14, 15). Likewise, neosporosis in cattle has also been widely reported in China. A recent review showed that the average seroprevalence of *N. caninum* was 13.69% in cattle herds in China, and the prevalence of *N. caninum* infection in cattle has been reported in many provinces of China (16). But so far, information on *N. caninum* infection in cattle in Shanxi Province is very limited, only two articles mentioned that the seroprevalence of *N. caninum* in cattle in Shanxi Province was 23.08% (6/26). Since these two studies used a very small number of samples (*n* = 26) from Shanxi and were published in 2007 (17, 18), these preliminary results are not sufficient for the correct assessment of *N. caninum* infection in cattle in Shanxi Province. The latest data from the Shanxi Provincial Bureau of Statistics, China showed that approximately 1,174,300 cattle are raised in Shanxi Province (19). Considering the economic importance of neosporosis in cattle industry and the lack of specific drugs and vaccines against *N. caninum*, it is particularly important to conduct an epidemiological survey of *N. caninum* infections and assess the risk factors associated with its seroprevalence in cattle in Shanxi Province.

Therefore, in order to investigate the seroprevalence of *N. caninum* infection in cattle in Shanxi Province, the indirect enzyme linked immunosorbent assay (ELISA) was used to examine anti-*N. caninum* antibodies in cattle serum samples collected from 11 cities of Shanxi Province, and to analyze the potential risk factors of *N. caninum* infection, with the objective of providing baseline data for executing effective measures to prevent and control *N. caninum* infection in cattle in Shanxi Province.

## Materials and methods

### Investigation sites

**Figure 1:**
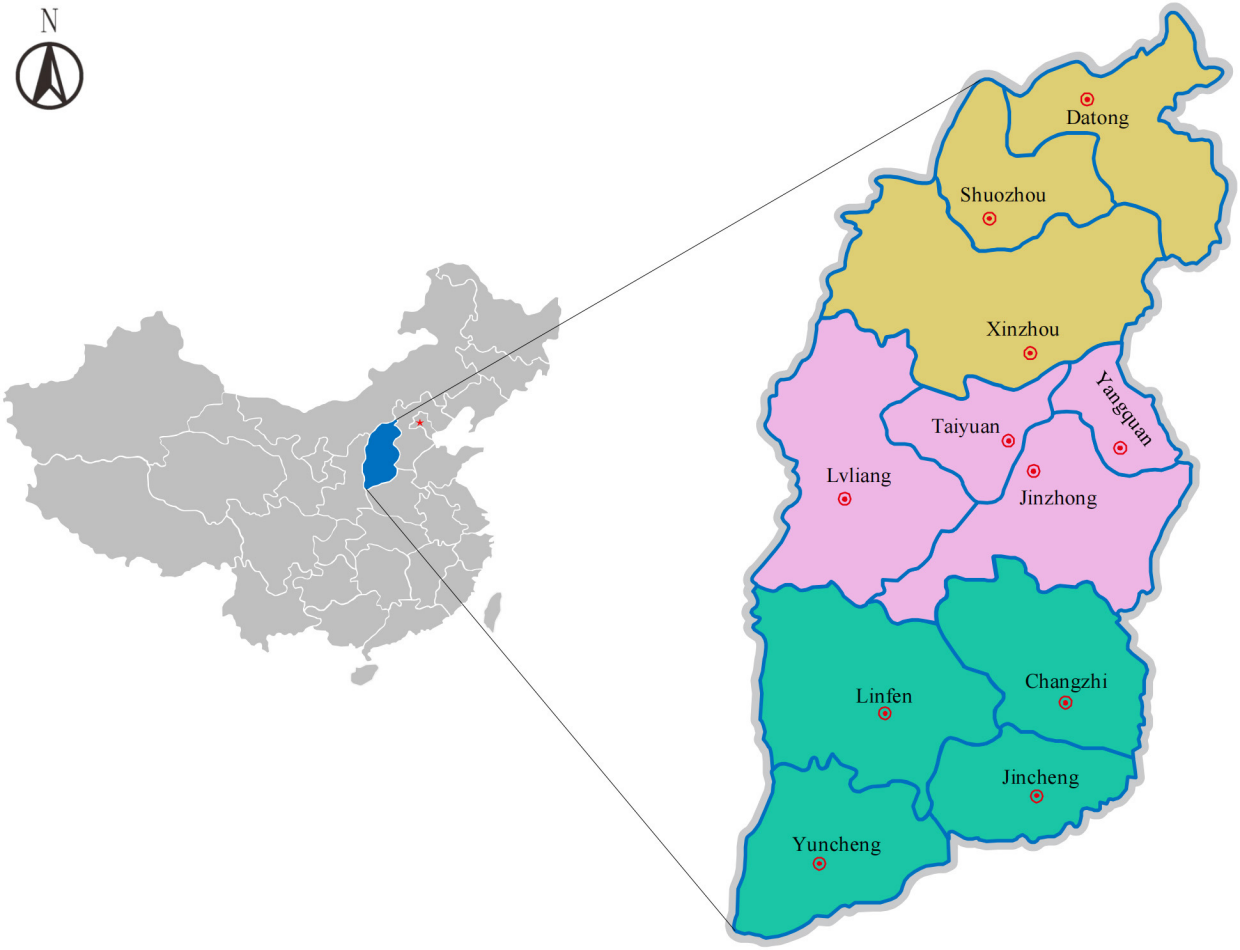
Map showing the geographical locations in Shanxi Province, north China. Yellow, pink and green colors represent the geographical location of Northern Shanxi (Datong, Shuozhou, Xinzhou), Central Shanxi (Taiyuan, Lvliang, Jinzhong, Yangquan) and Southern Shanxi (Changzhi, Jincheng, Linfen, Yuncheng), respectively. Red star mark: Beijing, the capital of China. Double circles: Eleven cities in Northern, Central and Southern Shanxi.

This study covered 40 cattle farms in 11 administrative cities in Shanxi Province, including three cities in Northern Shanxi, four cities in Central Shanxi and four cities in Southern Shanxi. Shanxi Province (34°36'-40°44'N, 110°15'-114°32'E) is located in north China and has a temperate continental monsoon climate with four distinct seasons and large temperature differences between the north and the south, with an average annual temperature of 3°C~14°C (Figure 1).

### Collection of samples

978 serum samples were randomly collected from cattle in 40 cattle farms in 11 cities in three geographical locations of Shanxi Province by blind sampling in November 2020; among which 267, 356 and 355 samples were collected from Northern Shanxi Province (Datong, Shuozhou and Xinzhou), Central Shanxi Province (Taiyuan, Lvliang, Jinzhong and Yangquan), and Southern Shanxi Province (Changzhi, Jincheng, Linfen and Yuncheng), respectively (Figure 1). Of the 978 cattle serum samples, 563 samples were from cattle in household cattle farms, 100 samples from cattle farming cooperatives and 315 samples from large-scale cattle farming companies. After collecting blood sample from each cattle, the serum was separated and transported to the laboratory, and stored at−40°C until analysis.

### Serological tests

The presence of *N. caninum*-specific IgG antibodies was detected using a commercial indirect ELISA kit (ID Screen^®^ *Neospora caninum* Indirect, Innovative Diagnostics, France), according to the manufacturer's specifications. Briefly, the reagents and serum samples were returned to room temperature (20°C), and then were shaken gently. The optical densities (ODs) of each sample were measured at 450 nm using an absorbance reader (CMax Plus, Molecular Devices, USA). The results were considered valid when the mean ODs of the positive control (OD_PC_) is >0.350, and the ratio between the OD_PC_ and the ODs of the negative control (OD_NC_) is >3. The value of S/P% per sample is calculated by [(OD sample-OD_NC_)/OD_PC_-OD_NC_] × 100%. Samples with S/P% ≥ 50% or ≤ 40% were considered positive or negative, respectively, and 40 < S/P% < 50% were considered as “suspicious samples” and were retested. The samples that were negative or positive after retesting were considered as negative or positive samples, respectively; while the samples that were still suspicious after retesting were considered as positive samples.

### Statistical analysis

Chi-square test was performed on the seroprevalence and potential risk factors (geographical location and management mode) of *N. caninum* infection in cattle herds in Shanxi Province using SPSS 26.0 software (Chicago, USA). The odds ratios (ORs) and their 95% confidence interval (95% CI) of each risk factor were analyzed in this study. *P* < 0.05 was considered statistically significant.

## Results

### Seroprevalence

In this study, 133 (13.60%, 95% CI = 11.45–15.75) of the examined 978 cattle serum samples from the 40 cattle farms were detected to be positive for anti-*N. caninum* antibodies by ELISA, with seroprevalence in different cities ranging from 0 to 78.89% (Table 1). Among the 133 positive cattle serum samples, 47 samples were from Northern Shanxi (17.60%, 95% CI = 13.03–22.17), 86 samples were from Central Shanxi (24.16%, 95% CI = 19.71–28.60), and 0 sample was from Southern Shanxi (0%); 54 samples were from household cattle farms (9.59%, 95% CI = 7.16–12.02), 0 sample was from cattle farming cooperatives (0%), and 79 samples were from large-scale cattle farming companies (25.08%, 95% CI = 20.29–29.87; Table 2).

**Table 1:** The seroprevalence of *N. caninum* in cattle in different cities of Shanxi Province.

**Geographical**	**City**	**No**.	**No**.	**Prevalence (%)**
**location**		**examined**	**positive**	**(95% CI)**
Northern Shanxi	Datong	88	29	32.95 (23.13–42.78)
	Shuozhou	89	18	20.22 (11.88–28.57)
	Xinzhou	90	0	0
Central Shanxi	Taiyuan	90	71	78.89 (70.46–87.32)
	Lvliang	90	0	0
	Jinzhong	87	4	4.60 (0.20–9.00)
	Yangquan	89	11	12.36 (5.52–19.20)
Southern Shanxi	Changzhi	90	0	0
	Jincheng	90	0	0
	Linfen	85	0	0
	Yuncheng	90	0	0
Total		978	133	13.60 (11.45–15.75)

**Table 2:** Analysis of the related variables of *N. caninum* infection in cattle in Shanxi Province.

**Variable**	**Categories**	**No. examined**	**No. positive**	**Prevalence (%)**	***P*-value**	**OR**
				**(95% CI)**		**(95% CI)**
Geographical location	Northern Shanxi	267	47	17.60 (13.03–22.17)	< 0.001	Reference
	Central Shanxi	356	86	24.16 (19.71–28.60)		1.49 (1.00–2.22)
	Southern Shanxi	355	0	0		–
Management mode	Household cattle farms	563	54	9.59 (7.16–12.02)	< 0.001	Reference
	Cattle farming cooperatives	100	0	0		–
	Large-scale cattle farming companies	315	79	25.08 (20.29–29.87)		3.16 (2.16-4.61)
Total		978	133	13.60 (11.45–15.75)		

### Risk factors

Regarding the risk factors associated with *N. caninum* seroprevalence in cattle in Shanxi Province, the results of the statistics are summarized in Table 2. Geographical location (*P* < 0.001) and management mode (*P* < 0.001) were revealed as risk factors for *N. caninum* infection in cattle herds in Shanxi Province. Considering the geographical locations of the cattle farms, the seroprevalence of *N. caninum* in cattle was higher in Central Shanxi than in Northern Shanxi and the difference between them was statistically significant (OR = 1.49; *P* < 0.001). Notably, cattle herds in Southern Shanxi were free from *N. caninum* infections; even after excluding Southern Shanxi, the seroprevalence of *N. caninum* in cattle was higher in Central Shanxi than in Northern Shanxi and the difference between them was statistically significant (OR = 1.49; *P* < 0.05; Supplementary Table 1). When considering the different management modes of the cattle farms, the seroprevalence of *N. caninum* was higher in cattle in large-scale cattle farming companies than in household cattle farms and the difference between them was statistically significant (OR = 3.16; *P* < 0.001). After excluding cattle farming cooperatives, the prevalence of *N. caninum* was higher in cattle in large-scale cattle farming companies than in household cattle farms and the difference between them was extremely significant statistically (OR = 3.16; *P* < 0.001; Supplementary Table 1). In addition, to explore the relationship between regions and management modes, the seroprevalence for the three management modes between different cities in each region was calculated in Table 3. The results showed that the *N. caninum* seroprevalence was higher in cattle in large-scale cattle farming companies in Northern Shanxi and Central Shanxi, while cattle in household cattle farms had a higher prevalence if they were in Taiyuan, Central Shanxi, and lower in other household cattle farms; while cattle farming cooperatives were unique to Central Shanxi and all had zero *N. caninum* seroprevalence (Table 3).

**Table 3:** The seroprevalence of *N. caninum* in cattle for the three management modes between different cities in each region in Shanxi Province.

**Geographical**	**City**	**Household cattle**			**Cattle farming**			**Large-scale cattle**		
**location**		**farms**			**cooperatives**			**farming companies**		
		**No**.	**No**.	**Prevalence**	**No**.	**No**.	**Prevalence**	**No**.	**No**.	**Prevalence**
		**examined**	**positive**	**(%)**	**examined**	**positive**	**(%)**	**examined**	**positive**	**(%)**
Northern Shanxi	Datong	0	–	–	0	–	–	88	29	32.95
	Shuozhou	59	2	3.39	0	–	–	30	16	53.33
	Xinzhou	90	0	0	0	–	–	0	–	–
Subtotal		149	2	1.34	0	–	–	118	45	38.14
Central Shanxi	Taiyuan	50	39	78.00	0	–	–	40	32	80.00
	Lvliang	10	0	0	80	0	0	0	–	–
	Jinzhong	87	4	4.60	0	–	–	0	–	–
	Yangquan	49	9	18.37	20	0	0	20	2	10
Subtotal		196	52	26.53	100	0	0	60	34	56.67
Southern Shanxi	Changzhi	30	0	0	0	–	–	60	0	0
	Jincheng	90	0	0	0	–	–	–	–	–
	Linfen	8	0	0	0	–	–	77	0	0
	Yuncheng	90	0	0	0	–	–	0	–	–
Subtotal		218	0	0	0	–	–	137	0	0
Total		563	54	9.59	100	0	0	315	79	25.08

## Discussion

*N. caninum* has been discovered for more than 30 years since the late 1980s (20). Due to its global distribution and efficient transmission, it seriously affects the cattle industry and leads to significant economic losses. Therefore, the prevention and control of *N. caninum* in cattle is very important. Vaccination has been recognized as the most desirable option to control the disease in cattle herds, however, there are no commercially available vaccines for the prevention of *N. caninum* (21). Therefore, the impact of the disease can only be reduced through biosecurity and management measures, and differentiating between infected and uninfected animals becomes the basis for disease management. Many diagnostic methods have been developed, such as PCR assays for detection of parasite DNA and serological tests for specific antibodies (8). *N. caninum* infection is usually diagnosed by serology in live cattle, indirect diagnoses through serological testing have great advantages, and ELISAs are used for high-throughput screening (11). Previous studies have shown that serological detection of *N. caninum* infection is accurate in examining tachyzoite-specific antibodies in cattle, the commercially available kits also have good test agreement and share high performance in terms of specificity and sensitivity (11, 22). This study therefore investigated the seroprevalence of *N. caninum* infection in cattle in Shanxi Province using a commercial indirect ELISA kit.

The seroprevalence of *N. caninum* in cattle varies considerably between different countries, as well as different regions and cattle farms in the same country; for example, the seroprevalence of *N. caninum* in Serbia and Iranian cattle herds was 7.20 and 23.60%, respectively (23, 24). The seroprevalence of *N. caninum* in cattle herds detected in different regions of Brazil ranged from 9.1 to 97.2% (25, 26). Mongolia and Vietnam border on the north and south of China, respectively. In Mongolia, the overall seroprevalence of *N. caninum* in cattle was 26.2%, sex was revealed as an important risk factor of *N. caninum* infection, and the *N. caninum* seroprevalence may be affected by climate and geographical conditions (15). In Southern Vietnam, the *N. caninum* seroprevalence varied between 16 and 53% in the state herds, and the prevalence of *N. caninum* was higher in the herds that had imported cows than in the herds that only had locally bred cows (27). In China, the average seroprevalence of *N. caninum* was 13.69% in cattle herds, and the presence or absence of pregnancy and the number of pregnancies is considered as risk factors (16). In the present survey, the average seroprevalence of *N. caninum* in cattle herds in Shanxi was 13.60% detected by ELISA, which was similar to the average seroprevalence in China. By comparing seroprevalence of *N. caninum* in cattle in Shanxi Province with that in neighboring provinces, we found that the seroprevalence of *N. caninum* in cattle in Shanxi Province was similar to that in Inner Mongolia Autonomous Region (IMAR) (15.88%) (28), but lower than that in Henan Province (41.20%) (29) and Hebei Province (37.34%) (30). Differences in seroprevalence of *N. caninum* may be related to specific characteristics of each region, such as climatic conditions, production systems, the presence of definitive hosts and herds health management, or to different study designs and diagnostic methods (31, 32).

The distance between the Northern and Southern Shanxi Province is more than 680 kilometers, with the large temperature differences and different local conditions and customs, which may be related to different *N. caninum* seroprevalence among cattle herds. This study showed that the seroprevalence of *N. caninum* in cattle in different cities of Shanxi Province varied from 0% in six cities to 32.95% in Datong in Northern Shanxi and 78.89% in Taiyuan in Central Shanxi. The geographical location was revealed as a risk factor for *N. caninum* infection in cattle in Shanxi Province (*P* < 0.001; Table 2). Studies have shown that sex, age, whether or not the cattle are pregnant, and the management mode of the farm are potential risk factors for *N. caninum* seroprevalence in cattle (16, 33). Unfortunately, the collection of cattle serum samples in the present study was done by blind sampling, thus data about the sex and age of the sampled cattle, and whether or not the cattle sampled was pregnant, were not available; so we explored whether management mode was a risk factor for *N. caninum* infection in cattle in Shanxi Province.

The results showed that cattle in household cattle farms (9.59%, 95% CI = 7.16–12.02) and large-scale cattle farming companies (25.08%, 95% CI = 20.29–29.87) had a higher seroprevalence of *N. caninum* than that in cattle farming cooperatives (0%). Compared to household cattle farms, the risk of *N. caninum* infection was more than three times higher (OR = 3.16, 95% CI = 2.16–4.61) for cattle in large-scale cattle farming companies. These results suggested that the management mode was a risk factor for *N. caninum* infection in cattle in Shanxi Province (*P* < 0.001; Table 2). Cattle in large-scale cattle farming companies may have higher *N. caninum* prevalence and wide distribution if sporulated oocysts are present in the breeding environment due to high breeding density, intensive management and mode of transmission routes (horizontal or vertical transmission). Therefore, we suggest that large-scale cattle farming companies should optimize the breeding environment, eliminate positive cattle in time and block its transmission in the herds. While household cattle farms are profit-oriented, the farm owners will be more responsible in the breeding process. However, household cattle farms have certain disadvantages in declaring subsidies and other aspects due to their small scale, so it may be difficult to carry out intervention measures due to limited funding, thus leading to the presence of *N. caninum* infection in cattle in household cattle farms, but the prevalence is not high and varies among household cattle farms. In addition, cattle farming cooperatives are favorably supported by governments of different levels with higher subsidy funds, thus the animal feeding management, medication and immunization procedures are more formal, which may be the reason for the low prevalence of *N. caninum* infection in cattle in cattle farming cooperatives.

Interestingly, by comparing the seroprevalence of *N. caninum* in cattle in three management modes in different cities of different regions (Table 3), we found that even though cattle in Central Shanxi (24.16%) has a higher seroprevalence of *N. caninum* than in Northern Shanxi (17.60%), most of the positive serum samples were from either household cattle farms or large-scale cattle farming companies from Taiyuan (71/86, 82.56%), where a large population (approximately 5 million), a booming pet market and ubiquitous presence of stray dogs may be responsible for the high rate of *N. caninum* infection in local cattle herds. Among the Northern Shanxi, the seroprevalence of *N. caninum* was higher in cattle in large-scale cattle farming companies, accounting for 95.74% (45/47) of the *N. caninum*-positive cattle serum samples in the whole Northern Shanxi, which may be due to that Datong and Shuozhou directly border the Inner Mongolia grassland, and its living habits are closer to the IMAR. IMAR is a pastoral area with a large number of shepherd dogs, so it is easier for *N. caninum* to spread in cattle herds, and the seroprevalence of *N. caninum* in IMAR cattle herds was 15.88% (28). Therefore, we suggest that the prevention and control of *N. caninum* on cattle farms in Taiyuan, Datong and Shuozhou should be strengthened and the government should increase the proportion of cattle farming cooperatives. In addition, future large-scale investigations should be conducted in cattle in Southern Shanxi to reveal the prevalence of *N. caninum* in cattle, so as to lay the foundation for the control of *N. caninum* on cattle farms.

## Conclusion

The average seroprevalence of *N. caninum* in cattle in Shanxi Province was 13.60% (133/978), and geographical location and management mode were revealed as the risk factors related to *N. caninum* infection in cattle herds in Shanxi Province, north China. This was the first report of a large-scale investigation of the seroprevalence and risk factors for *N. caninum* in cattle herds in Shanxi Province, which not only extended the *N. caninum* seroprevalence data in cattle, but also provided baseline data to prevent and control *N. caninum* infection in cattle in Shanxi Province.

## Data availability statement

The original contributions presented in the study are included in the article/Supplementary material, further inquiries can be directed to the corresponding authors.

## Ethics statement

The animal study was reviewed and approved by the Experimental Animal Ethics Committee of Shanxi Agricultural University (Approval No. 2019IACUCSXAU002A01).

## Author contributions

BT and H-YS conceived and designed the experiments. HC and W-BZ performed the experiments, analyzed the data, and wrote the paper. YW, Y-PL, and QL participated in the collection of serum samples. W-WG and QL participated in the implementation of the study. H-YS, QL, BT, and X-QZ critically revised the manuscript. All authors have read and approved the final version of the manuscript.

## Funding

Project support was provided by Fund for Shanxi 1331 Project (Grant No. 20211331-13), the Special Research Fund of Shanxi Agricultural University for High-level Talents (Grant No. 2021XG001), and the Yunnan Expert Workstation (Grant No. 202005AF150041).

## Conflict of interest

The authors declare that the research was conducted in the absence of any commercial or financial relationships that could be construed as a potential conflict of interest.

## Publisher's note

All claims expressed in this article are solely those of the authors and do not necessarily represent those of their affiliated organizations, or those of the publisher, the editors and the reviewers. Any product that may be evaluated in this article, or claim that may be made by its manufacturer, is not guaranteed or endorsed by the publisher.
