# Supplementary material for: Seroprevalence of Neospora caninum infection and associated risk factors in cattle in Shanxi Province, north China
Source: Front Vet Sci. 2022 Nov 29;9:1053270. doi: 10.3389/fvets.2022.1053270 (PMC9744922; doi:10.3389/fvets.2022.1053270)
Supplement: Supplementary file 1 [file Table_1.DOCX]

**SUPPLEMENTARY TABLE 1** **|** Analysis of the related variables of *N. caninum* infection in cattle in Shanxi Province, China (Excluding zero-infection groups)

| **Variable** | **Categories** | **No. examined** | **No. positive** | **Prevalence (%)**  **(95% CI)** | ***P*-value** | **OR**  **(95% CI)** |
| --- | --- | --- | --- | --- | --- | --- |
|  |  |  |  |  |  |  |
| Geographical location | Northern Shanxi | 267 | 47 | 17.60 (13.03-22.17) | < 0.05 | Reference |
|  | Central Shanxi | 356 | 86 | 24.16 (19.71-28.60) |  | 1.49 (1.00-2.22) |
| Management mode | Household cattle farms | 563 | 54 | 9.59 (7.16-12.02) | < 0.001 | Reference |
|  | Large-scale cattle farming companies | 315 | 79 | 25.08 (20.29-29.87) |  | 3.16 (2.16-4.61) |
